# Motivators of impulsivity to smoke waterpipe tobacco among Nigerian youth who smoke waterpipe tobacco: the moderating role of social media normalisation of waterpipe tobacco

**DOI:** 10.1186/s12889-022-13386-4

**Published:** 2022-05-27

**Authors:** Agatha Oluwafunmilayo Adu, Nurzali Ismail, Shuhaida Md. Noor

**Affiliations:** School of Communication, Universiti Sains Malaysia, Penang, Malaysia

**Keywords:** Waterpipe tobacco smoking, Impulsivity, Social media, Normalisation, Youth, University, Nigeria

## Abstract

**Background:**

Impulsivity is a formidable cause of waterpipe tobacco smoking among youth, however, it is understudied among African youth. Using PRIME behavioural theory, this study aimed to develop a model that examines the motivators of impulsivity to smoke waterpipe tobacco in linkage to the moderating role of social media normalisation of waterpipe tobacco, specifically among youth in Nigeria who smoke waterpipe tobacco.

**Methods:**

Data were drawn from 695 respondents who smoke waterpipe tobacco across six Nigerian universities in the South-West zone using the chain-referral sampling procedure. Descriptive analyses of the obtained data were carried out using the Statistical Package for Social Sciences (SPSS) version 25. The constructs in the developed model were validated through Partial Least Squares Structural Equation Modelling (PLS-SEM) in SmartPLS version 3.

**Results:**

Among Nigerian youth who smoke waterpipe tobacco, intention (β = 0.442, *P* < 0.001) was the strongest motivator of impulsivity to smoke waterpipe tobacco as compared to positive evaluations (β = 0.302, *P* < 0.001). In addition, social media normalisation of waterpipe tobacco acted as a moderator that strengthened the relationship between intention and impulsivity (β = 0.287, *P* < 0.01), as well as, between positive evaluations and impulsivity (β = 0.186, *P* < 0.01) among youth.

**Conclusion:**

Intention greatly instigates Nigerian youth’s impulsivity to smoke waterpipe tobacco, and social media normalisation of waterpipe tobacco also considerably increases their impulsivity to smoke waterpipe tobacco. Youth-focused educational waterpipe tobacco cessation-oriented programmes that utilise diverse constructive-based learning approaches like illustrative learning and counselling, can help to enlighten and encourage Nigerian youth on the importance of shunning the desirability to smoke waterpipe tobacco.

## Introduction

Over the years, the use of tobacco has remained a worldwide hazardous issue causing a mortality rate of over 7 million annually [1]. Consumption of tobacco is often by various means such as cigarette, cigar, chewing, snuff, snus, waterpipe, amongst others [2]. Among these forms of tobacco use, waterpipe is perhaps the most dangerous as it is mostly assumed to be negligibly harmful, which has contributed to its increasing popularity especially among youth [3]. In North America for instance, waterpipe tobacco smoking (WTS) reportedly increased among Canadian (42%) and American (2%) youth between the year 2012 and 2014 [4, 5]. Also in the Middle East, WTS is reportedly high among Iranian (26.6%) and Palestinian (23%) youths [6, 7]. In Africa, although empirical data on WTS is still scant [8], studies have shown that, WTS is growing rapidly among youth in Rwanda (26.1%), Uganda (36.4%), and Nigeria (44%) [9–11].

Realistically, WTS should decrease rather than increase among youth as waterpipe tobacco (WT) is notably more harmful compared to other methods of tobacco use [12]. Risk of respirational damage is higher among WT smokers. In addition to the smoke from the lit charcoal in the waterpipe, they also inhale greater amount of tobacco smoke due to the lengthy propensity of smoking sessions, often lasting up to an hour or more, unlike cigarette smoking that can be done within five minutes [13]. The unsanitary act of sharing waterpipe hose during smoking sessions is also related to the transmission of communicable diseases like tuberculosis and meningitis [14]. More recently, the deadly Corona Virus Disease 2019 (COVID-19) which is elicited by a group of immensely transferrable viruses known as Severe Acute Respiratory Syndrome Coronavirus-2 (SARS-CoV-2), is also associated with WTS [15]. A COVID-19 infected WT smoker can infect others as the exhalation of tobacco smoke is mingled with fluid from the salivary gland [16]. Thus, youth’s unceasing impulsivity to smoke WT remains a public health concern that should be further comprehended.

Impulsivity is described as an uncontrollable nudge to carry out an action, such that when it arises, there is a high possibility for the associated action to immediately take place. As such, impulsive tobacco smoking can be difficult to control [17]. One major motivating factor of youth’s impulsivity to smoke WT is intention, also referred to as willingness [18]. Supporting instance is a study in Canada that found 18% propensity to smoke WT among youth who had the willingness to do so [19]. Positive evaluations, also known as positive beliefs or feelings [20], is another influential motivator of youth’s impulsivity to smoke WT [21]. A separate study in Jordan revealed that, the vulnerability to smoke WT among youth is strongly associated with positive evaluations such as the sweetness of its tobacco flavours like chocolate and strawberry amongst others, as well as the ability to smoke it within a social gathering [22].

Furthermore, social media normalisation of waterpipe tobacco seems to have the ability to heighten the relationship between youth’s related intention, positive evaluations, and impulsivity [23]. Normalisation in the tobacco context symbolises the situation whereby many people in the society now consider an unhealthy behaviour like tobacco smoking as a normal and social behaviour rather than a harmful one that should be eliminated [24]. As such, social media normalisation of WT is a concept that explains; majority of the social media content related to WT in the format of text, pictures and videos frequently encourage rather than discourage its usage [25]. Information pertaining to the use of waterpipe tobacco on social media like Twitter, Facebook, and Instagram is more pro-smoking than anti-smoking [26]. This implies that the characteristics of social media, which allows unrestrictive exposure to information, has exposed youth to a variety of information that are devoid of the harmful nature of WT [25]. This also takes into account that, youth are avid users of social media [27].

The objective of this study was to develop a model that investigates intention and positive evaluations as factors influencing impulsivity, while considering the moderating effect of social media normalisation in relation to WTS among youth in Nigeria. To achieve this study’s aim, we concentrated on the lens of PRIME (plans, responses, impulses, motives, evaluations) behavioural theory which explain that, actions that are unhealthy and addictive are heralded by an impulsiveness which is instigated by intention, positive evaluations, as well as outer environmental factors such as media information [28].

### Theoretical underpinning

Our theoretical underpinnings were built upon Robert West’s PRIME behavioural theory, which describes plans, responses, impulses, motives, and evaluations as related to addictive behaviour [29]. Diverging from other behavioural theories such as the Theory of Planned Behaviour and Theory of Reasoned Action that emphasize on intention [30], PRIME focuses on impulsivity and inhibition [28]. The PRIME perspectives explain that, impulsivity is the drive for an individual’s susceptibility to an addictive behaviour, while inhibition is the drive for an individual’s ability to refrain from an addictive behaviour [31]. Regarding impulsivity, an individual’s prior intent (or plan) to act on an addictive behaviour, encouragement from outer environmental or personal motivators, as well as, positive evaluations (or beliefs), can stimulate irresistible response to act on such behaviour [32]. However, in the presence of inhibition, an individual’s prior intent not to act on an addictive behaviour, discouragement from outer environmental or personal motivators, as well as, negative evaluations can generate the ability to resist the desirable urge to act on such behaviour [33]. In this regard, impulsivity is laced by irrationality, while inhibition is linked to rationality [34].

Past studies have indicated the applicability of inhibition based on PRIME’s perspectives to effective tobacco smoking cessation efforts [35–38]. Nevertheless, PRIME’s relevance to the understanding of impulsivity as related to tobacco smoking is less studied [30]. Considering that PRIME is a developing theory [39], empirical studies focusing on both inhibition and impulsivity are important to demonstrate the theory’s usefulness in understanding addictive behaviour like tobacco smoking. The present study formulated a predictive model which proposes that there is a positive link between impulsivity, intent (or plan), and positive evaluations (or beliefs), as indicated in the existing studies [7, 40]. Also, based on prior evidences [25, 41], we included social media normalisation of WT as an outer environmental factor that can moderate the relationship between intention, positive evaluations, and impulsivity among youth.

### Hypotheses development

We developed hypotheses for intention to smoke waterpipe tobacco, positive evaluations of waterpipe tobacco, and social media normalisation of waterpipe tobacco.

#### Intention to smoke waterpipe tobacco (ITSW)

Intention refers to the willingness to carry out an action [42]. In the context of this study, intention refers to the willingness to smoke WT which could generate an impulsiveness to smoke it. Studies have shown that, readiness to experiment with WT constitutes the urge to smoke among youth, hence leading to impulsiveness to smoke due to inability to resist the urge [9, 10, 19, 40, 43]. In Nigeria, willingness is also a key factor in the growing tendency to smoke WT among youth, particularly those in universities [11]. As such, we assumed that, there is a positive connection between intention and impulsivity among Nigerian youths who tend to smoke WT. Hence, the following hypothesis was proposed:

##### H1

Intention to smoke waterpipe tobacco is positively related to impulsivity among Nigerian youth who have the tendency to smoke waterpipe tobacco.

#### Positive evaluations of waterpipe tobacco (PEOW)

Positive evaluations entail beliefs, feelings, or notions that depict something in an advantageous manner [44]. In the context of waterpipe tobacco, positive notions that WT is not causing significant harm and addiction persists among youth, thereby instigating their continuous urge to smoke it [7]. Youth’s tendencies to smoke WT are further stimulated by positive feelings such as, the activity is sociable, entertaining and provide tasty flavours [45]. Positive feelings about WT also relate to the Nigerian context, as many youth consider it as a fun activity [46]. Consequently, we proposed that, there is a positive association between positive evaluations and impulsivity among Nigerian youth who tend to smoke WT and hypothesised that:

##### H2

Positive evaluations of waterpipe tobacco is positively related to impulsivity among Nigerian youth who have the tendency to smoke waterpipe tobacco.

#### Social media normalisation of waterpipe tobacco as a moderator (SMNW)

Social media normalisation of waterpipe tobacco indicates, the enormous depiction of WT on social media platforms like Facebook, Twitter, Instagram and YouTube, as an acceptable behaviour rather than a detrimental one [47]. The frequent positive illustration of WT on social media is concerning. While harmful potential is often acknowledged in social media messages related to other tobacco smoking methods [41], those associated with WT frequently describe it as a safer tobacco smoking alternative [25]. In fact, most of the texts, pictures, videos, and other graphics related to WT on social media are reportedly representative of waterpipe as being socially acceptable, fun, interesting, entertaining, enjoyable and attractive [48, 49]. Studies also have shown that exposure to pro-smoking social media messages increases the youth’s tendency to smoke tobacco [23, 50], especially among those who hold positive beliefs and are willing to experiment with it [51]. Therefore, it was considered that, the positive relationship between intention, positive evaluations, and impulsivity is more intense among youth with high exposure to pro-smoking social media messages that relate to WT. Consequently, we hypothesised that:

##### H3a

Social media normalisation of waterpipe tobacco moderates the positive relationship between intention and impulsivity, such, the tendency to smoke waterpipe tobacco is stronger among Nigerian youth with high exposure to pro-smoking social media messages relating to waterpipe tobacco.

##### H3b

**Fig. 1:**
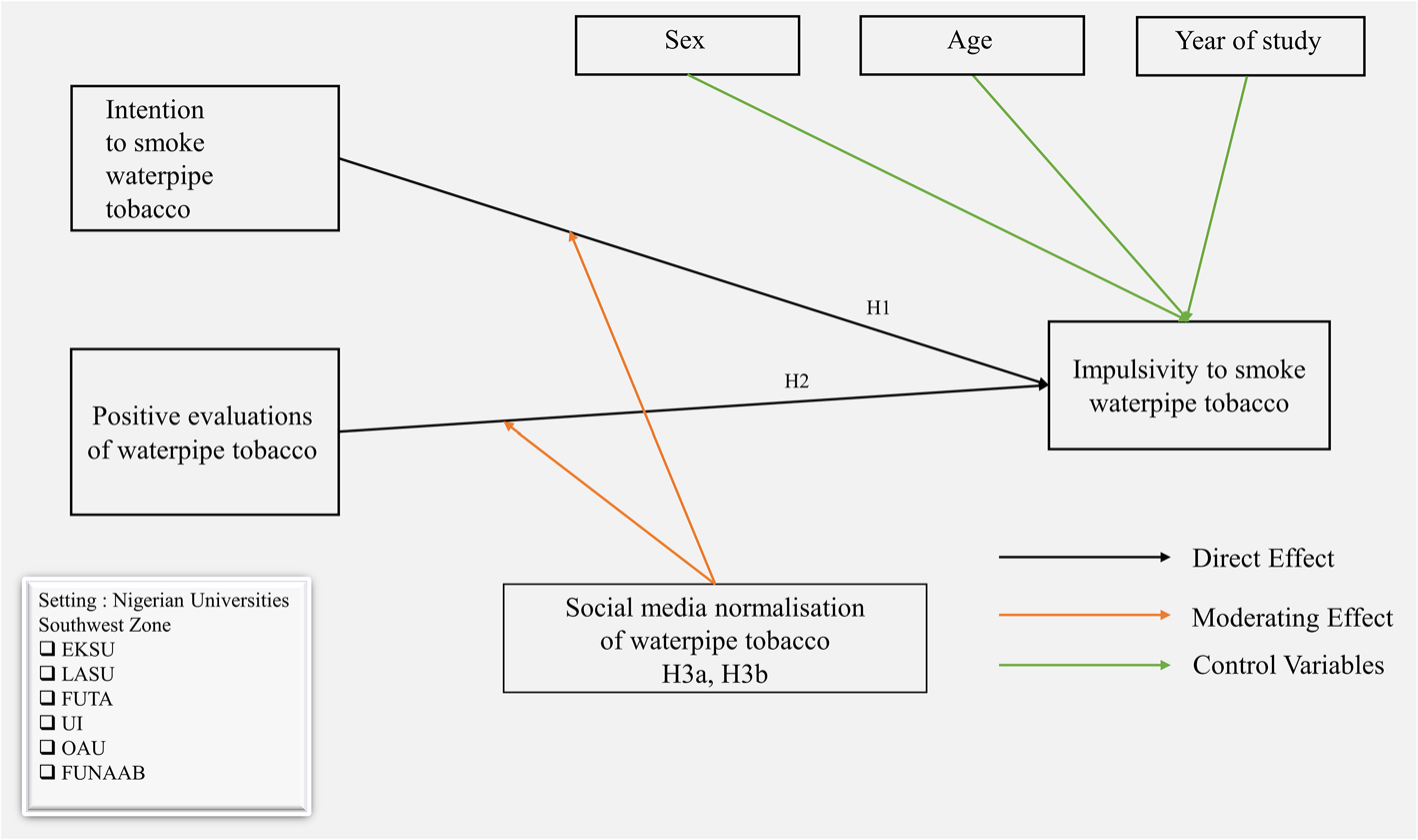
A research model for Nigerian youth’s impulsivity to smoke waterpipe tobacco

Social media normalisation of waterpipe tobacco moderates the positive relationship between positive evaluations and impulsivity, such, the tendency to smoke waterpipe tobacco is stronger among Nigerian youth with high exposure to pro-smoking social media messages relating to waterpipe tobacco (Fig. 1).

## Methodology

### Research setting and design

This study adopted a cluster-based cross-sectional design using survey. Considering that many Nigerian youth in universities consider WT as a normal and social activity [11], we surveyed youth between ages of 18-35 who; smoke WT, are social media users and university students in the South-West of Nigeria, where tobacco smoking is common [52]. Specifically, one university was selected in each of the six states in the South-West of Nigeria namely; the Ekiti State University Ado-Ekiti [EKSU] (Ekiti State); Lagos State University [LASU] (Lagos State); Federal University of Technology Akure [FUTA] (Ondo State); University of Ibadan [UI] (Oyo State); Obafemi Awolowo University Ile-Ife [OAU] (Osun State); and the Federal University of Agriculture Abeokuta [FUNAAB] (Ogun State) [53]. Respondents were excluded if: (1) they have not smoked WT at least once, (2) not male or female between the ages of 18-35, (3) not current students in any of the selected universities, (4) not social media users, (5) not willing to participate.

In terms of sample size, we dealt with a heterogeneous group, as well as, a hidden population due to the specific focus on Nigerian youth who smoke WT [54, 55]. We calculated 110 respondents for each of the selected universities using a confidence level of 99% and a margin of error of 5% [56]. As for the actual data collection procedure, we took into account the likelihood for missing data, as well as, youth who do not smoke WT. Hence, the required sample size (n = 110) for each university was increased by 40% as recommended by the literature [57]. Therefore, the actual number of respondents who participated in the survey was 154 in each of the six selected universities, and 924 respondents overall.

The survey questionnaires were physically administered and the chain-referral non-probability sampling procedure was utilised for respondents’ recruitment. To facilitate field work, a student was recruited as a field assistant in each of the six universities. They were extensively briefed on the study’s criteria. To ensure that only WT smokers were recruited, tracking was performed through the following affirmation in the questionnaire, “*Never smoked waterpipe.”* Regarding social media usage, respondents were required to affirm the specific type(s) of social media that they use in the questionnaire. The age category in the questionnaire was strictly within the ages of 18-35 to ensure that all respondents fulfilled the study’s requirement.

The data was collected between May – August 2021. To ensure prevention from COVID-19, all field assistants were required to use face masks during interactions with the respondents, maintained social distance, and regularly used hand sanitiser. In addition, field assistants were required to bring along face masks for respondents’ usage. Hand sanitisers and face masks were provided by the researchers. Out of the 924 respondents, 188 (20.3%) were excluded due to not being WT smokers, while 41 (4.4%) responses were excluded due to missing data. Researchers excluded responses containing missing data if relative percentage was less than 15% and the useable data was adequate enough [58].

### Constructs and measures

This study had four constructs, two independent variables (intention to smoke WT—ITSW, positive evaluations of WT—PEOW), one dependent variable (impulsivity to smoke WT—IMP), and one moderating variable (social media normalisation of WT—SMNW). All items were rated on a seven-point Likert scale ranging from “1= *strongly disagree* to 7= *strongly agree*,” and all were reflective measures. The four items for intention and the nine items for impulsivity were adopted from prior studies [59, 60]. The items for positive evaluations of WT and social media normalisation of WT were developed using a construct development process [61]. First, we interviewed six youth volunteers (3 males, 3 females) who smoke WT and are social media users. They were recruited for the interview through one of the author’s contacts who works in a Nigerian university. Anonymity was requested and guaranteed.

For the positive evaluations of WT construct, the interviewees were asked to describe their preferences for WT as opposed to other tobacco smoking methods. Only one of the participants (female) reported that, she enjoyed smoking WT even though she was aware that it is harmful to health. Other interviewees reported that, WT is less harmful and addictive, and they had favourable opinion on it. As for the social media normalisation of WT construct, the interviewees were asked to confirm whether they had come across social media messages that promote the use of WT and to give their opinion on the influence of such messages based on their own experience. One of the interviewees indicated that she only came across such messages a few times. The remaining five interviewees reported that they regularly encountered such messages. Four interviewees (3 males, 1 female) indicated that they liked the visual and interesting nature of social media WT messages. This led to social media engagement behaviours such as like, comment and share.

Based on the interviews and prior studies; positive evaluations of WT [22, 62] and social media normalisation of WT [51, 63], eight initial items for both constructs were generated. Next, five expert panel on youth behavioural studies and social media were consulted pertaining to the constructs. Based on the consultation with the expert panels, items for positive evaluations of WT and social media normalisation of WT were changed to nine and six respectively.

In attempt to minimise ambiguity of the survey, a preliminary study was conducted with 70 youth respondents who smoke WT in a Nigerian university. Although 50 respondents are sufficient for pilot testing [64], this study surveyed 70 youth respondents, an increase by 40% due to the possibility of the respondents who may not have the experience of smoking WT and non-response to the questionnaire [57]. Questionnaires were physically administered using the chain-referral procedure. Out of the 70 questionnaires distributed, 53 (response rate 75.7%) were useable. The final questionnaire is presented in Table 1.

**Table 1:** Constructs and items

Constructs	Code	Items
**Intention to smoke waterpipe** [59]	ITSW1	I intend to continue smoking waterpipe.
	ITSW2	I will buy waterpipe if I happened to see it in a store, café, lounge, or restaurant.
	ITSW3	I will actively seek out waterpipe in a store, café, lounge, or restaurant to purchase it.
	ITSW4	I will patronise waterpipe as a tobacco smoking product.
**Positive evaluations of waterpipe (Self-developed)**	PEOW1	Waterpipe smoking is attractive.
	PEOW2	Waterpipe smoking is fun.
	PEOW3	Waterpipe smoking is relaxing.
	PEOW4	Waterpipe smoking is romantic.
	PEOW5	Waterpipe smoking offers enjoyable fruity flavours.
	PEOW6	Waterpipe smoking is more acceptable in the society compared to other methods of tobacco smoking.
	PEOW7	Waterpipe smoking is less addictive compared to other methods of tobacco smoking.
	PEOW8	Waterpipe smoking is less harmful compared to other methods of tobacco smoking.
	PEOW9	In general, I have favourable opinion on waterpipe smoking.
**Impulsivity to smoke waterpipe** [60]	IMP1	I often smoke waterpipe spontaneously.
	IMP2	“Just do it” describes the way I smoke waterpipe.
	IMP3	I often smoke waterpipe without thinking.
	IMP4	“I see waterpipe, I smoke it” describes me.
	IMP5	“Smoke waterpipe now, think about it later” describes me.
	IMP6	Sometimes I feel like smoking waterpipe on the spur of the moment.
	IMP7	I smoke waterpipe according to how I feel at the moment.
	IMP8	I carefully plan most of my waterpipe smoking sessions. (R)
	IMP9	Sometimes, I am a bit reckless about waterpipe smoking.
**Social media normalisation of waterpipe (Self-developed)**	SMNW1	In the past 6 months, I have regularly seen pictures, videos, or other pro-smoking content promoting the use of waterpipe on social media (e.g., Facebook, Instagram, YouTube, Twitter, Tik-Tok, etc.).
	SMNW2	In the past 6 months, I have regularly commented positively, reposted, or clicked like on pictures, videos, or other pro-smoking content promoting the use of waterpipe on social media (e.g., Facebook, Instagram, YouTube, Twitter, Tik-Tok, etc.).
	SMNW3	Pro-smoking waterpipe messages on social media makes me feel like waterpipe smoking is normal.
	SMNW4	Pro-smoking waterpipe messages on social media makes me want to smoke waterpipe.
	SMNW5	Pro-smoking waterpipe messages on social media can make people choose waterpipe as a tobacco smoking alternative.
	SMNW6	Pro-smoking waterpipe messages on social media can make people want to smoke waterpipe.

*R refers to reverse coded items

### Data analyses method

Statistical Package for Social Sciences (SPSS) version 25 was used to analyse the descriptive segment for frequencies, percentages, mean, and standard deviation of the participants’ characteristics and variables distribution. Common method bias (CMB) was also assessed using SPSS. Partial least squares structural equation modelling (PLS-SEM) was applied using SmartPLS version 3 to assess the significance of the model constructs through measurement model, structural model, and moderator analyses [65]. We also tested the collinearity among the constructs using SmartPLS. In order to estimate the significance of the relationship between dependent and independent variables, it was proposed that other variables referred to as confounders to be controlled in the analysis [66]. If the path value between the independent variables and the dependent variable was greater than the path value between the control variables and the dependent variable, this indicates that the independent variables to be the most influential factors in the model [67]. Using SmartPLS, we therefore controlled sex, age, and year of study as they could potentially influence the impulsivity to smoke WT among university students [21].

## Results

In total, 695 (response rate 75.2%) questionnaires were useable for the analysis. A sample size of 695 generated a power of over 95%, as such, our sample was adequate to generate confident outcomes [68]. Table 2 shows that the number of female respondents (53.2%) slightly outweighed male respondents (46.8%), indicating that female youth who smoke WT were higher than males. Respondents were categorised into three age groups: 18-20 (22.3%), 21-26 (62.6%), and 27-35 (15.1%). Most of the respondents were students in LASU (17.4%), while the lowest number of respondents were from FUNAAB (16.3%). Majority of the respondents were in their second year of study (26.0%) while the smallest number were postgraduate students (3.5%). Respondents were asked to rate the regularity with which they generally smoke WT, and those who frequently smoked it (41.9%) were slightly higher than occasional smokers (38.4%), while a smaller number indicated that they had smoked it at least once (19.7%). Similarly, majority of the respondents reported that they had smoked WT between 1-5 years (96.8%), compared to a small number of respondents who had smoked it between 6-10 years (3.2%).

**Table 2:** Participants’ profile

Characteristics	Frequency	Percentage (%)
**Sex**		
Male	325	46.8
Female	370	53.2
**Age**		
18-20	155	22.3
21-26	435	62.6
27-35	105	15.1
**University**		
FUTA	114	16.4
EKSU	117	16.8
FUNAAB	113	16.3
OAU	116	16.7
UI	114	16.4
LASU	121	17.4
**Year of study**		
First year	172	24.7
Second year	181	26.0
Third year	168	24.2
Fourth year	122	17.6
Fifth year	28	4.0
Postgraduate studies	24	3.5
**Rate of WTS**		
At least once	137	19.7
Occasional smoker	267	38.4
Frequent smoker	291	41.9
**Length of WTS**		
1-5 years	673	96.8
6-10 years	22	3.2
**Social media usage**		
Between 1-5 hours daily	238	34.2
Between 5-10 hours daily	342	49.2
More than 10 hours daily	115	16.5
**Type of social media**		
Facebook	134	19.3
Twitter	110	15.8
Instagram	147	21.2
Tik-Tok	125	18.0
YouTube	93	13.4
Others	86	12.4
**Ethnicity**		
Yoruba	244	35.1
Igbo	185	26.6
Hausa	137	19.7
Others	129	18.6

Majority of the respondents used social media between 5-10 hours daily (49.2%), while a smaller number used of it for more than 10 hours daily (16.5%). Instagram was the most used social media platform (21.2%), followed by Facebook (19.3%), Tik-Tok (18.0%), Twitter (15.8%), YouTube (13.4%), and others (12.4%). In the “others” category, the respondents specified the platforms as WhatsApp, Telegram, and Snapchat. In term of their ethnicities, majority of the respondents were Yoruba (35.1%), followed by Igbo (26.6%), Hausa (19.7%), and others (18.6%). Participants who selected “others” specified their ethnicities as Fulani, Ebira, Edo, Tiv, Idoma, Ibibio, Itsekiri, Efik, Ijaw and Urhobo.

### Common method bias and collinearity

CMB was assessed using Harman’s single factor test. The single factor was less than 50%, which signifies that, there was no single factor that explained the majority of the variance in our data [69]. In addition, we also checked for collinearity by examining the variance inflation factor (VIF) of the constructs. The results showed that, all constructs did not exceed the boundary of 5 (see Table 3) [54]. Hence, CMB and collinearity posed no concern in this study [70].

**Table 3:** Construct reliability, composite reliability, AVE, and VIF values

Code	Outer loading	CA α	CR	AVE	VIF	***M***	***SD***
ITSW1	0.828	0.890	0.924	0.753	1.897	4.13	1.78
ITSW2	0.883					3.96	1.86
ITSW3	0.886					3.73	1.85
ITSW4	0.872					3.96	1.87
PEOW1	0.878	0.943	0.952	0.688	1.959	4.72	1.87
PEOW2	0.859					5.01	1.74
PEOW3	0.901					4.83	1.83
PEOW4	0.819					4.49	1.83
PEOW5	0.830					5.13	1.73
PEOW6	0.787					4.95	1.74
PEOW7	0.800					4.77	1.71
PEOW8	0.790					4.73	1.72
PEOW9	0.790					4.63	1.73
IMP1	0.833	0.937	0.948	0.674	–	4.39	1.78
IMP2	0.836					4.55	1.78
IMP3	0.851					4.46	1.83
IMP4	0.891					4.30	1.89
IMP5	0.868					4.47	1.81
IMP6	0.847					4.48	1.71
IMP7	0.845					4.62	1.73
IMP8	0.519					5.13	1.48
IMP9	0.836					4.25	1.88
SMNW1	0.785	0.911	0.927	0.680	1.050	5.36	1.48
SMNW2	0.866					5.07	1.67
SMNW3	0.902					5.15	1.63
SMNW4	0.889					5.12	1.62
SMNW5	0.741					5.35	1.49
SMNW6	0.750					5.43	1.39

### Measurement model

Except for IMP8 which had a loading of 0.519, the study’s indicator loadings, composite reliability, and Cronbach’s alpha values were above 0.70 [71]. It was recommended that an indicator loading >0.40 but <0.70 should be retained if the Average Variance Extracted (AVE) is >0.50 [65], consequently, IMP8 was retained since its related AVE exceeded 0.50 (see Table 3). For discriminant validity, all values were below 0.90 as recommended [72] (see Table 4).

**Table 4:** Discriminant validity: Heterotrait-Monotrait (HTMT)

Variables	IMP	ITSW	PEOW	SMNW
IMP				
ITSW	0.707			
PEOW	0.634	0.743		
SMNW	0.211	0.113	0.202	

### Structural model

**Fig. 2:**
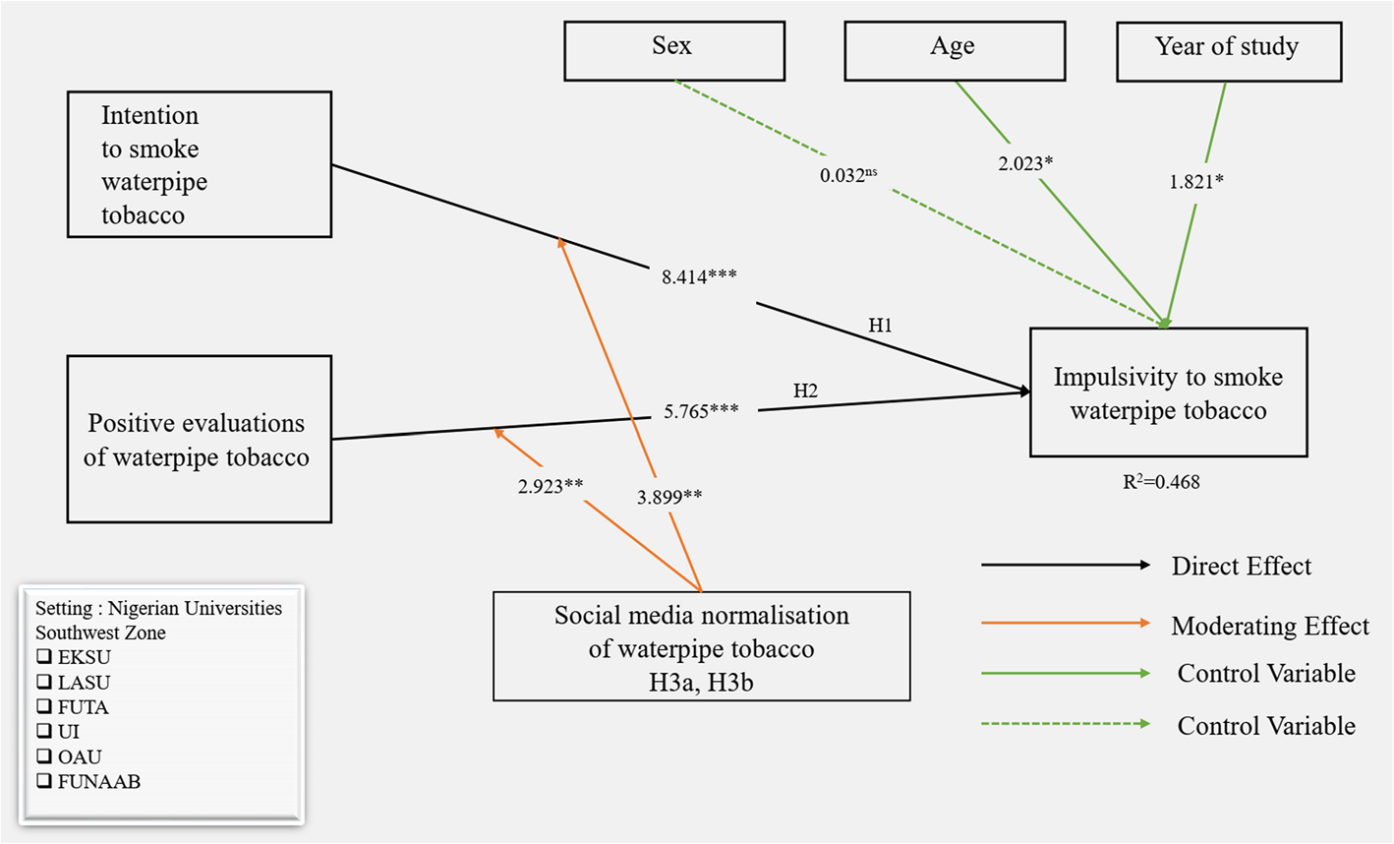
Structural model for Nigerian youths impulsivity to smoke waterpipe tobacco. *Significant at *p* < 0.05, **at *p* < 0.01, ***at *p* < 0.001, and ns-not significant

We tested the significance of the study’s model using a 5000 bootstrapping procedure at 5% significance level (one-tailed) [71]. Table 5 and Fig. 2 shows that intention (β = 0.442, *P* < 0.001) and positive evaluations (β = 0.302, *P* < 0.001) positively predicted impulsivity in relation to WT. Therefore, H1 and H2 were supported in our study. Effect sizes for H1 (f^2^ = 0.194) and H2 (f^2^ = 0.091) were acceptable based on the rule of thumb of 0.02, 0.15, and 0.35 as small, medium, and large respectively [73]. Also, the predictive relevance (Q^2^) of our model was 0.312 > 0, indicating that the model has excellent predictive relevance [68]. Finally, our model explained 46.8% of the variance (R^2^) in youth’s impulsivity to smoke WT. A variance that is equal to or > 0.10 is considered to be adequate [74]. As such, the predictors in our model substantially explained the dependent variable.

**Table 5:** Structural model results

Code	Hypotheses	β	SE	Q^**2**^	f^**2**^	Decision
H1	ITSW - > IMP	0.442	0.052	0.312	0.194	Supported***
H2	PEOW- > IMP	0.302	0.051	–	0.091	Supported***
H3a	ITSW*SMNW- > IMP	0.287	0.042	–	0.136	Supported**
H3b	PEOW*SMNW- > IMP	0.186	0.061	–	0.112	Supported**

*Significant at *p* < 0.05, **at *p* < 0.01, and ***at *p* < 0.001

### Testing the moderator

**Fig. 3:**
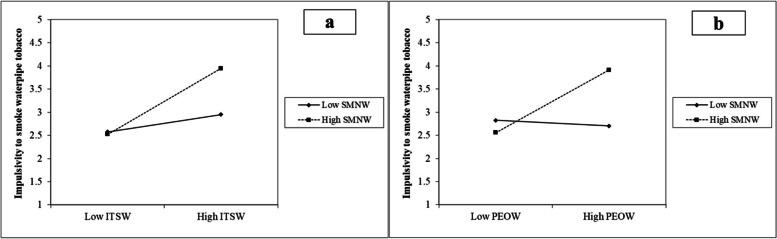
Moderation effect of SMNW. **a** Effect on the relationship between ITSW and impulsivity to smoke waterpipe tobacco. **b** Effect on the relationship between PEOW and impulsivity to smoke waterpipe tobacco

The results showed that social media normalisation of WT moderates the relationship between intention and impulsivity (β = 0.287, *P* < 0.01), as well as, between positive evaluations and impulsivity (β = 0.186, *P* < 0.01). As evidenced by the dotted line which is on a higher level compared to the solid line, Fig. 3 a and 3b indicate that social media normalisation of WT considerably strengthens the relationship between intention and impulsivity, as well as, between positive evaluations and impulsivity. Based on the effect size (f^2^) in moderation analysis which is 0.025, 0.01, and 0.005 for large, medium, and small respectively [75], it can be assumed that, the f^2^ values of the moderating effects, H3a (0.136) and H3b (0.112) indicate large moderation effects (see Table 5).

## Discussion of findings

As for the main effects in our study, results showed that both intention and positive evaluations predicted impulsivity in relation to WTS among youth. This suggests that willingness and positive feelings prompt youth’s impulsiveness to smoke WT. This particular finding also proposes that, the influential power of intention and positive evaluations on impulsivity to smoke WT among Nigerian youth is similar to other African countries [9, 10, 76] and non-African countries [5, 7, 77, 78] where studies have reported similar findings. Our results also revealed that, intention has more effect on impulsivity to smoke WT compared to positive evaluations. This finding is consistent with the previous research which suggested that, intention is a formidable predictor of youth’s likelihood to smoke WT [79]. Therefore, willingness creates an acceptability that strongly contribute to the tendency to smoke WT among youth [40].

Regarding the moderation effects, we found that, the relationship between intention and impulsivity to smoke WT is stronger among youth with high exposure to pro-smoking social media messages related to WT. This particular finding suggests that, social media messages that encourage the use of WT have the potential to increase the youth’s desirability to smoke it. This result is similar to prior research which reported that, there is a positive link between social media pro-smoking messages and tobacco smoking tendency among youth [23]. In addition, it was found that, social media normalisation of WT moderates the relationship between positive evaluations and impulsivity to smoke WT among youth. This finding proposes that, positive beliefs, feelings, or notions about WT appear to be stronger among youth who have high exposure to social media pro-smoking messages related to WT. This outcome is comparable to previous findings, indicating that there is a significant connection between social media and positive expressions of tobacco smoking among youth [51].

Fig. 3a and b further demonstrated the relevance of our study’s findings on the moderation effect. According to the findings, social media normalisation of WT strengthens the influence of intention and positive evaluations on impulsivity to smoke WT among youth who already have high intention and positive evaluations. This suggests that, there is a crossover effect of social media among youth. It takes into account the influence of social media pro-smoking messages that increase impulsivity among youth who have high intention and positive evaluations to smoke [50, 80]. In this instance, pro-smoking social media messages are normalising WT in a way that may compound youth’s urge to smoke it, and increase their impulse [81]. The findings of this study emphasised the importance of Article 13 of the Framework Convention on Tobacco Control (FCTC), that urges the prohibition of tobacco advertising, promotion, and sponsorship (TAPS) [82]. Considering that Nigeria is one of the FCTC’s participating countries [83], our study proposed that, there is a need to create measures to aid the implementation of the framework. This includes implementing paragraph 4 of Article 13 in FCTC which specifically proposes a ban on TAPS in both traditional and internet-based media [82]. This can contribute towards minimising the growing influence of social media normalisation of WT among youth.

The findings of this study also suggested that, there is a need to minimise youth’s accessibility to WT, as their tendency to smoke appears to increase. Studies have indicated that, raising the minimum legal age for access is one of the ways to minimise the accessibility to WT among youth [84, 85]. In many countries, the minimum legal age for access to tobacco is 21-year-old [86]. However, some African countries seem to adhere to lower legal age of tobacco access. For instance, the legal age of tobacco access in Malawi and Egypt is 14-year-old [84], while in Nigeria, is 18-year-old [87]. While some may argue that an 18-year-old youth is old enough to smoke if he or she desires, it is recommended that, the legal age is raised to 21-year-old. This considers the detrimental effects of smoking [84]. Raising the legal age for tobacco smoking may serve good purpose for Nigerian youth, particularly in the light of the findings of this study which suggests growing inclination to smoke WT among youth.

## Conclusion

The findings of this study suggested that intention and positive evaluations are factors that instigate the impulsivity to smoke WT among Nigerian youth. Greater effect of intention was found on impulsivity to smoke WT as compared to positive evaluations among youth. The results also showed that, social media normalisation of WT increases the effect of intention and positive evaluations on youth’s impulsivity to smoke WT. Although age and year of study have impact on the youth’s impulsivity to smoke WT, the path value of the independent variables (intention, positive evaluations) suggested that they exhibit the greatest influence on youth’s impulsivity to smoke WT. These outcomes have important theoretical and practical implications as discussed in the following sections.

### Theoretical and empirical implications

Our study contributed to the advancement of the PRIME theory. Previous studies mostly utilised PRIME from its perspective of inhibition, by adopting it in the assessment of tobacco smoking cessation [35–38]. Hence, by expounding its perspectives of disinhibition as related to impulsivity to smoke WT, this study has responded to the existing gap to demonstrate the applicability of a developing theory like PRIME in addressing addictive behaviour [30]. In the African context which empirical research on WT, especially among youth is scarce [8], this study contributed to knowledge building. Prior studies on WT within the Nigerian context only provided preliminary understanding, particularly in regard to youth [11, 88, 89]. Hence, this study addressed the need by providing empirical findings on WTS among youth in Nigeria. This study also responded to the call to theoretically explicate Nigerian youth’s tendency to smoke WT [90]. The items developed for the measurement of the two constructs (positive evaluations of WT, social media normalisation of WT) can be adopted and extended in future research.

### Practical implications

We posited that, although governmental policies that restrict WTS may discourage its usage among youth, enactment of policies may not be adequate enough to curb youth’s impulsivity to smoke WT. Thus, we suggested that the Nigerian government should collaborate with other stakeholders such as the National Universities Commission (NUC), conventional media professionals, and public health professionals, to introduce and implement youth-focused educational WT cessation programmes that utilise diverse constructive-based learning approaches like counselling, as well as, cooperative and illustrative learning. Such programmes can help youth to feel a sense of enlightenment and encouragement, rather than forceful expectancies divulged through policies. We also suggested that, digital-based platforms should be incorporated in WT related cessation efforts as this may help to neutralise the influential role of social media normalisation of WT among Nigerian youth.

### Limitations and suggestion for further studies

Even though this study has broadened our understanding of youth’s impulsivity related to WT within the understudied context like Nigeria, it has some limitations that should be acknowledged and addressed. This study focused only on Nigerian youth who are current university students in the South-West of the country. This was due to the rapid rise of WTS among youth in universities [11], and the difficulty to locate current WT smokers in Nigeria [91]. In this study we only surveyed youth in the South-West of Nigeria where the use of tobacco is reportedly popular among youth [52]. Future studies should consider participation of respondents from other geopolitical zones and to also include youth who are not in universities. Future studies should also consider addressing youth’s impulsivity related to WT using other measures including peer and family influence either as direct effects or mediators.

We acknowledged that some data and analysis that would have provided further insights for our study were not included in the survey. For instance, the findings on whether the respondents were daily or non-daily smokers would have increased the understanding of youth’s characteristics. In addition, items addressing the frequency with which the respondents may have smoked WT within a specific period e.g., in the last 30 days, would have provided a clearer perspective on the rate of WTS among youth. We also did not adjust the clusters which could have contributed to the comprehension of the similarities and dissimilarities between the groups in the study. It was proposed that, future research to explore these perspectives to further enrich knowledge on youth WTS. Future studies should also consider taking a closer look into the actual WTS behaviour rather than focusing only on impulsivity. This can help to aid understanding of the specific smoking patterns among youth including daily smoker, weekly smoker and other patterns.

Our study was also limited by the use of cluster-based cross-sectional design survey. The interpretation of the findings from survey was limited to the period within which the survey was conducted. This can be addressed by performing longitudinal studies that investigate related factors over a longer period of time with opportunity to generate a more in-depth data and generalisable outcomes.

## Data Availability

Datasets are available from the corresponding author on reasonable request.

## Abbreviations

WT: Waterpipe Tobacco
WTS: Waterpipe Tobacco Smoking
COVID-19: Corona Virus Disease 2019
ITSW: Intention to Smoke Waterpipe
PEOW: Positive Evaluations of Waterpipe
SMNW: Social Media Normalisation of Waterpipe
IMP: Impulsivity to Smoke Waterpipe
PRIME: Plans, Responses, Impulses, Motives, Evaluations
PLS-SEM: Partial Least Squares Structural Equation Modelling
SPSS: Statistical Package for Social Sciences
CMB: Common Method Bias
VIF: Variance Inflation Factor
AVE: Average Variance Extracted
CA: Cronbach Alpha
CR: Composite Reliability
M: Mean
SD: Standard Deviation
SE: Standard Error
EKSU: Ekiti State University
LASU: Lagos State University
FUTA: Federal University of Technology Akure
UI: University of Ibadan
OAU: Obafemi Awolowo University
FUNAAB: Federal University of Agriculture Abeokuta
NUC: National Universities Commission
